# Management of the Acute Scrotum in a District General Hospital: 10-Year Experience

**DOI:** 10.1100/tsw.2009.37

**Published:** 2009-04-28

**Authors:** Lukas Tajchner, John O. Larkin, Michael G. Bourke, Ronan Waldron, Kevin Barry, Paul W. Eustace

**Affiliations:** Department of Surgery, Mayo General Hospital, Castlebar, Co. Mayo, Ireland

**Keywords:** acute scrotum, scrotal exploration, testicular torsion

## Abstract

The acutely painful scrotum is a common urologic emergency. The primary objective of management is to avoid testicular loss. This requires a high index of clinical suspicion and prompt surgical intervention. In our series conducted between January 1996 and December 2005, 119 patients (age range: 4–62 years) underwent emergency operative exploration for acute scrotal pain. The most common finding was torted cyst of Morgagni (63/119, 52.9%), followed by testicular torsion (41/119, 34.4%). The majority of testicular torsions occurred in the pubertal group (22/41, 53.6%). Only one patient in this group had an unsalvageable testis necessitating orchidectomy, a testicular loss rate in torsion of 2.4%. There were no postoperative wound infections or scrotal haematomas. Testicular salvage depends critically on early surgical intervention, so the delay incurred in diagnostic imaging may extend the period of ischaemia. Furthermore, all radiological investigations have a certain false-negative rate. We advocate immediate surgical exploration of the acute scrotum. We report a low orchidectomy rate (2.4%) in testicular torsion.

## INTRODUCTION

The acutely painful scrotum is a common urologic emergency. The differential diagnosis rests between testicular torsion and all other causes of pain in the scrotum, for testicular torsion is the true emergency. The primary objective of management of the acute scrotum is to avoid testicular loss. This requires a high index of clinical suspicion and prompt surgical intervention. Many centres report high specificities for both radioisotope scanning and colour Doppler ultrasound studies to investigate acute scrotal pain[1]. However, testicular salvage is critically dependent on early surgical intervention, so the delay incurred in diagnostic imaging studies may serve to extend the period of testicular ischaemia and jeopardise the prospect of testicular salvage in the setting of spermatic cord torsion. Apart from the inherent delay, all radiological investigations have a certain false-negative rate. For this reason, some authors advocate emergency surgical exploration in all cases of acute scrotal pain.

We reviewed our unit's experience with emergency exploration of the acutely painful scrotum.

## METHODS

The records of all patients who underwent emergency exploration for acute scrotal pain at the Department of Surgery, Mayo General Hospital, during a 10-year period between January 1996 and December 2005, were reviewed retrospectively. The age, duration of symptoms, operative findings, morbidity rates, and orchidectomy rates were considered. Results were subdivided according to whether patients were prepubertal, pubertal, or postpubertal (an arbitrary designation made according to age under 12, between 12 and 16, or over 16, respectively). Patients were identified from the operating theatre register and admissions office records. Urine cultures were found to be negative in all patients. Patients with positive urine cultures, with or without scrotal swelling, who were thought clinically to have epididymo-orchitis had a scrotal ultrasound to confirm the diagnosis and were treated nonoperatively with antibiotics. These patients (n = 27) were excluded from this study.

At operation, access to the testicle was through a midline scrotal incision. A testis was deemed viable on its appearance at the time of surgery after conventional procedures to optimise and assess blood flow (warm saline packs or bleeding from an incision into the tunica albuginea). Orchidectomy was performed if an ischaemic testis was deemed unsalvageable or was necrotic. Where a testis was deemed viable, orchidopexy was performed by suture fixation. Torted cysts of Morgagni were excised. Contralateral orchidopexy was routinely performed. A scrotal support was routinely applied to prevent postoperative haematoma formation.

All patients with a true torsion were followed up in the outpatient department at 1 week, 6 weeks, and 3 months postexploration.

## RESULTS

In the 10-year period between January 1996 and December 2005, 119 patients (age range: 4–62 years) underwent emergency operative exploration for acute scrotal pain. Forty-four patients were prepubertal (aged under 12), 47 were pubertal (aged between 12 and 16), and 28 were postpubertal (aged over 16). Overall, the most common finding was torsion of a cyst of Morgagni (63/119, 52.9%), with testicular torsion the next most common finding (41/119, 34.4%). The majority of testicular torsions occurred in the pubertal group (22/41, 53.6%). Only one patient in this group had an unsalvageable testis at exploration, necessitating orchidectomy for established necrosis. Symptoms had been present for over 24 h in this patient. This was the only orchidectomy required out of all 41 torsions in our series, a rate of 2.4%. There was no scrotal abscess found at exploration in any patient. There were no postoperative wound infections and no patient had a scrotal haematoma that required exploration.

- *Prepubertal group* — The most common diagnosis in the prepubertal group was torsion of a cyst of Morgagni (28/44, 63%). There were 11 spermatic cord torsions (11/44, 25%), one case of epididymitis, and four normal examinations. There were no twisted undescended testes in this group (Fig. 1).
- *Pubertal group* — The most frequent findings in the pubertal group were torsion of the spermatic cord (22/47, 46.8%) and torsion of a cyst of Morgagni (21/47, 44.6%), one case of epididymitis, one exploration carried out for trauma, and two normal examinations (Fig. 2).
- *Postpubertal group* — In the postpubertal group, torsion of a cyst of Morgagni was again the most common intraoperative finding (14/28, 50%), with eight torsions of the spermatic cord (8/28, 28.6%), one epididymitis, one exploration for scrotal trauma, and four normal examinations (Fig. 3).

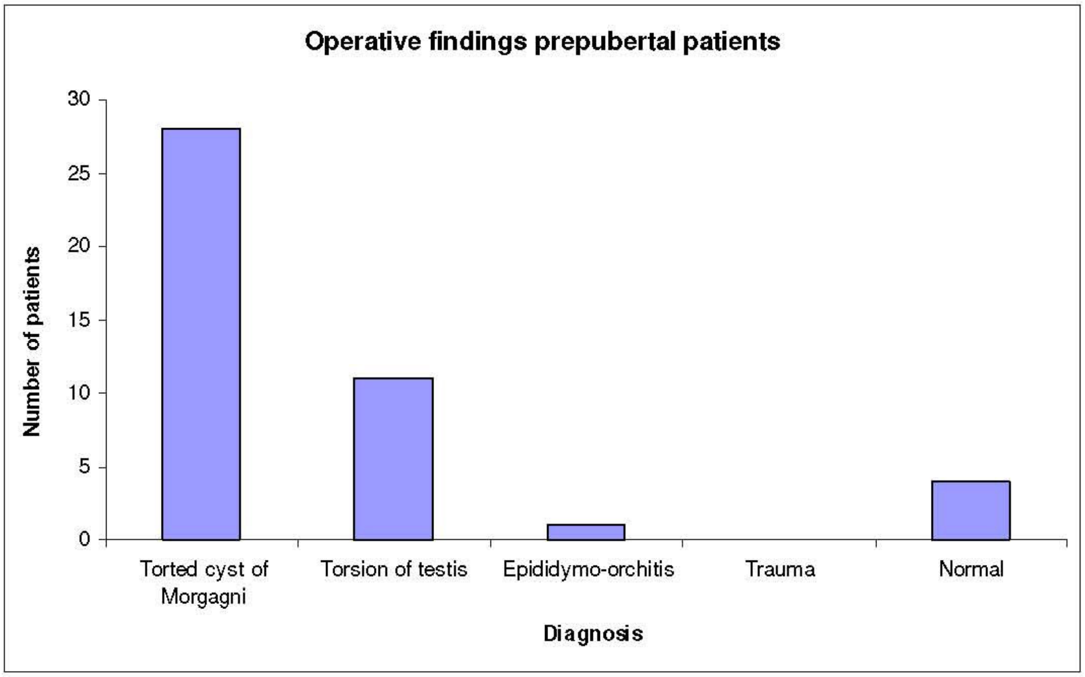

**FIGURE 1.** Operative findings in scrotal exploration in prepubertal patients (n = 44).

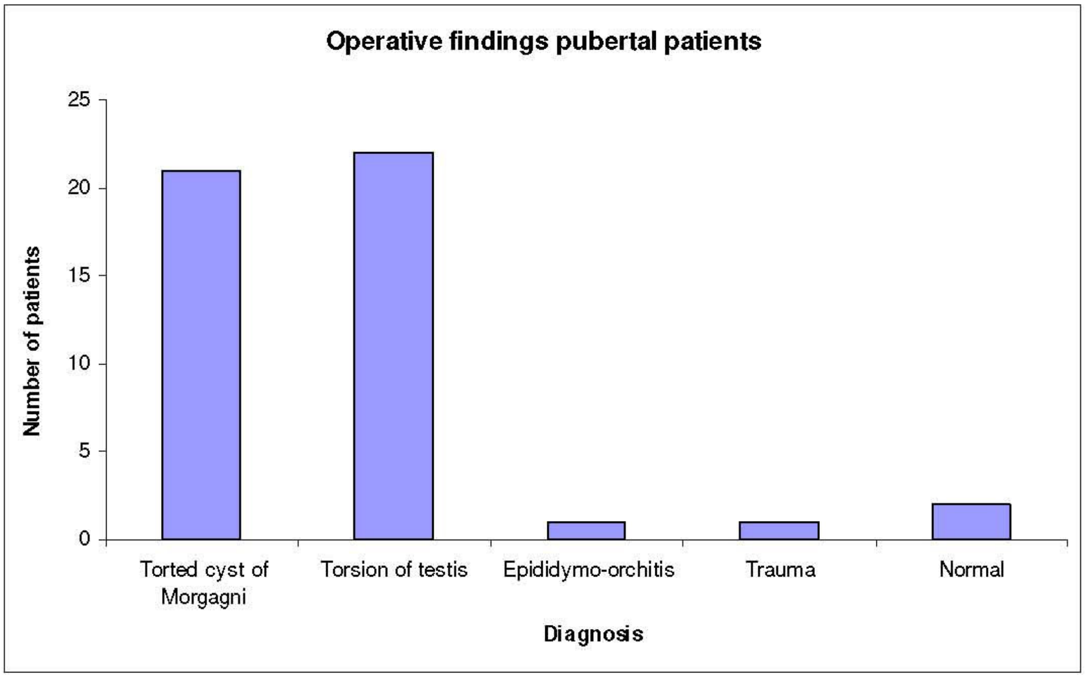

**FIGURE 2.** Operative findings in scrotal exploration in pubertal patients (n = 47).

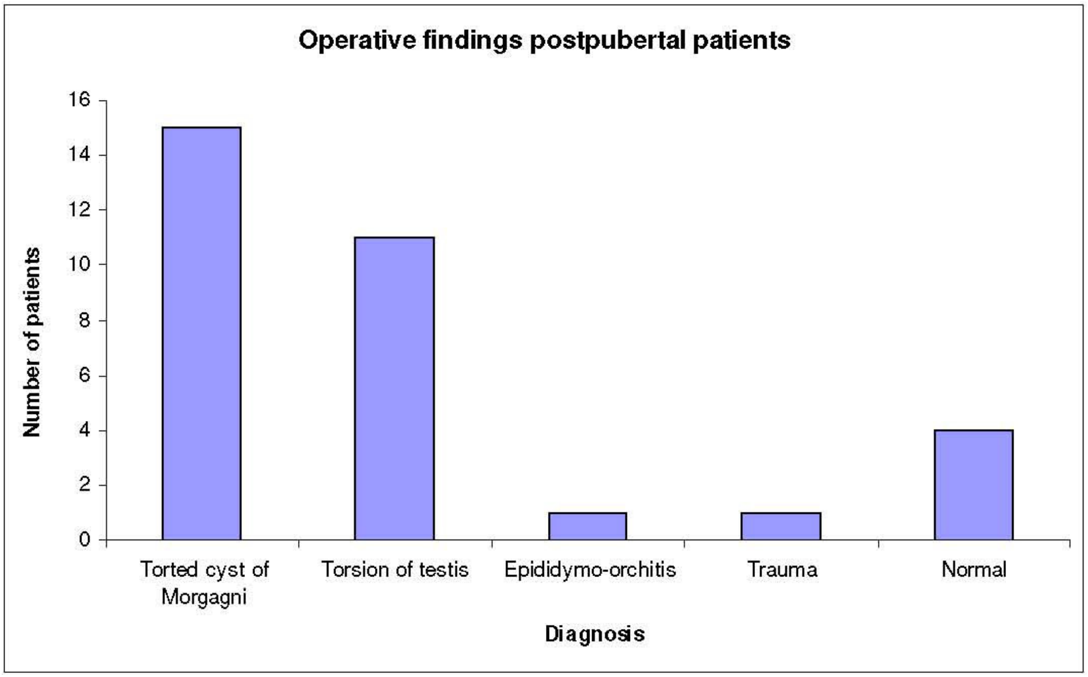

**FIGURE 3.** Operative findings in scrotal exploration in postpubertal patients (n = 28).

## DISCUSSION

The presenting symptoms of all acute scrotal pathologies overlap, and examination of the testicle can be hindered by oedema and extreme tenderness[2]. Where a diagnosis of testicular torsion cannot be excluded, it is generally agreed that emergency surgical exploration is mandatory. The policy in our unit is immediate surgical exploration of the acute scrotum, with limited recourse to radiological imaging. No avoidable testicular loss will occur if torsion is corrected while the testis is still salvageable. Consequently, our orchidectomy rate is low. There are several documented series that evaluate the diagnostic contribution of imaging studies in acute scrotal pain. The aim of such radiological investigations is to identify patients who do not have testicular torsion and may be managed conservatively. The specificity of radioisotope scanning has been reported to vary from 70–95.4%, with sensitivity of 78.6–85.7%[1,3]. For colour Doppler ultrasound scanning, the same authors reported specificity rates of 76.9–97% and sensitivity of 78.6–80%.

These specificities and sensitivities are unacceptably low in respect of the devastating outcome of a false-negative result. Indeed, in one of these series, there were four torsions missed by both imaging modalities. These false-negative results were for patients who presented late, with massively swollen intrascrotal contents, and intense reactive scrotal wall erythema and oedema. For all four studies, the resultant reactive inflammatory hyperaemia from a necrotic testis was attributed as the reason for the false-negative result[1]. While inflammation may be confirmed if increased flow is observed on colour Doppler ultrasound, the observation of normal flow certainly does not exclude the possibility of acute spermatic cord torsion[4]. Indeed, it has been argued that patients with spermatic cord torsion with preserved testicular perfusion are at particular risk for missed torsion when the diagnosis of torsion is based solely on colour flow Doppler findings[5]. It is also accepted that intermittent torsion may account for equivocal results of imaging studies[1].

Testicular salvage is critically dependent on the interval between onset of pain and surgical intervention[1]. In one series of 209 scrotal explorations, the overall rate of testicular salvage was 75%. Relief of torsion within 4 h resulted in complete viability; up to 16 h, 89% of testes were salvaged, but this decreased to 25% after 16 h. However, the testis was preserved in three cases in which continuous pain had been present for more than 24 h and where the testis was still twisted at the time of surgery. These values for testicular viability confirm that surgery should always be an emergency procedure[2].

In a series of 238 patients, no patient for whom the interval between the onset of pain and presentation to hospital was greater than 48 h had a salvageable testis. The success rate was 50% among patients who presented between 6 and 48 h after the onset of pain[1].

Clearly, it is imperative that expedient surgical exploration be undertaken to maximise the chance of testicular salvage. A number of studies in the literature illustrate that if a policy of conservative management is adopted on the basis of clinical findings and symptom duration alone, a small but nevertheless significant number of torted testicles will be missed[2].

In patients for whom testicular necrosis is considered to be inevitable because of the duration of symptoms, there is an argument for not operating as an emergency. However, it is mandatory to perform at least a contralateral orchidopexy without undue delay to avoid the catastrophic risk of metachronous torsion and anorchia[6].

Although rare in the adult population, testicular torsion has been described in patients up to 69 years of age[7]. Studies looking specifically at patient age at diagnosis of intravaginal testicular torsion report that up to 39% of patients are over 21 years of age[2,8,9,10].

Transient episodes of testicular pain, with or without swelling, followed by spontaneous resolution, in young adolescent boys are suggestive of intermittent testicular torsion. On the basis of history alone, early orchidopexy is recommended to prevent testicular loss at a future episode of torsion[11,12,13]. Some authors have reported that 30–50% of patients with acute torsion described prior episodes of acute testicular pain that may have represented intermittent testicular torsion[12,14].

In addition to testicular loss, the presence of testicular torsion may indicate a poor prognosis for the contralateral testis; certainly, the risk of torsion in the latter is increased, with 30% in one study subsequently developing symptoms[15]. The risk of ipsilateral testicular atrophy has been shown to increase with the duration of torsion[16]. As such, while a testis may be deemed salvageable at operative exploration, the ischaemic insult may nonetheless impair or extinguish its subsequent spermatogenic function. We do not have follow-up data on testicular function in respect of fertility, but none of the patients in our series who had an orchidopexy performed following operative salvage of a torted testis had ongoing pain suggestive of ischaemic orchitis at subsequent outpatient follow-up at 1 week, 6 weeks, and 3 months after exploration.

Of note in our series, the incidence of torted cyst of Morgagni is significantly higher, at 52.9% overall, than is reported in the modern literature. This figure is surprisingly high, particularly so in the postpubertal group, where the figure is 50%. Many authorities recommend that a torted cyst of Morgagni should be managed conservatively. This may be feasible where radiology expertise is immediately available and imaging findings unequivocal. Some studies have shown that immediate scanning by a senior paediatric radiologist may obviate surgical exploration if direct and complete visualisation of a nontwisted spermatic cord is achieved[17].

However, clearly, where findings are not definitive, immediate exploration is indicated. Our policy has been to explore all of these cases surgically. Critics of this approach argue that exploration with normal findings (8.4% in our series) or in the setting of a torted cyst of Morgagni represents unnecessary surgery, exposing patients to the risk of surgical and anaesthetic morbidity. However, in our series, there was no postoperative morbidity and we feel this approach is justified by the low orchidectomy rate we report.

While these patients may have been managed conservatively in retrospect, operative exploration of the acute scrotum obviates the false-negative rate and inherent temporal delay of radiological investigation, and minimises the rate of testicular loss. To our knowledge, there has been no reported testicular morbidity after exploration or fixation of a normal testis[6].

## CONCLUSION

The primary objective of management of the acute scrotum is to avoid testicular loss. The likelihood of testicular salvage in torsion depends on the interval between onset of pain and surgical intervention. Surgical exploration undertaken without delay maximises the chance of testicular salvage in testicular torsion.
